# Matrix Vesicles as a Therapeutic Target for Vascular Calcification

**DOI:** 10.3389/fcell.2022.825622

**Published:** 2022-01-21

**Authors:** Tiantian Li, Hongchi Yu, Demao Zhang, Tang Feng, Michael Miao, Jianwei Li, Xiaoheng Liu

**Affiliations:** ^1^ Institute of Biomedical Engineering, West China School of Basic Medical Sciences & Forensic Medicine, Sichuan University, Chengdu, China; ^2^ Division of Oral & Craniofacial Health Sciences, University of North Carolina Adams School of Dentistry, Chapel Hill, NC, United States; ^3^ Department of Endocrinology and Metabolism, West China Hospital, Sichuan University, Chengdu, China

**Keywords:** vascular calcification, matrix vesicles, extracellular vesicles, exosomes, vascular smooth muscle cells

## Abstract

Vascular calcification (VC) is linked to an increased risk of heart disease, stroke, and atherosclerotic plaque rupture. It is a cell-active process regulated by vascular cells rather than pure passive calcium (Ca) deposition. In recent years, extracellular vesicles (EVs) have attracted extensive attention because of their essential role in the process of VC. Matrix vesicles (MVs), one type of EVs, are especially critical in extracellular matrix mineralization and the early stages of the development of VC. Vascular smooth muscle cells (VSMCs) have the potential to undergo phenotypic transformation and to serve as a nucleation site for hydroxyapatite crystals upon extracellular stimulation. However, it is not clear what underlying mechanism that MVs drive the VSMCs phenotype switching and to result in calcification. This article aims to review the detailed role of MVs in the progression of VC and compare the difference with other major drivers of calcification, including aging, uremia, mechanical stress, oxidative stress, and inflammation. We will also bring attention to the novel findings in the isolation and characterization of MVs, and the therapeutic application of MVs in VC.

## Introduction

Vascular calcification (VC) is a prominent clinical pathology of atherosclerosis, diabetes mellitus, hypertension, aging, and chronic kidney disease (CKD), resulting in abnormal calcium phosphate accumulation in the intimal and medial layers of the vessel wall (Xu et al., 2020). After vascular calcification, the stiffness of the vascular wall is increased, and the compliance is decreased, which results in myocardial ischemia, left ventricular hypertrophy, and heart failure (Nitta and Ogawa, 2015). At present, vascular calcification is still lacks effective treatment methods, and the pathogenesis mechanism remains unclear (Herrmann et al., 2020). Therefore, it is necessary to uncover its specific mechanisms and develop therapeutic strategies.

The phenotype switching of the VSMCs has been regarded as the principal driver in the calcification of intimal and medial layers. VSMCs undergo the phenotypic transformation from a differentiated “contractile” into a dedifferentiated “synthetic” proliferative phenotype in the process of vascular calcification. During this phenotypic switching, VSMCs show decreased expression of the contractile markers smooth muscle α-actin (α-SMA), smooth muscle 22α (SM22α), smooth muscle myosin heavy chain 11 (SM-MHC), CNN1, calponin, leiomodin, smoothelin (SMTN), myosin light chain (MYL) and an increase in synthetic marker S100A4, KLF4, vimentin, osteopontin (OPN) (Furmanik et al., 2020). The phenotypic switching VSMCs express higher osteoblast-like markers, such as runt-related transcription factor 2 (Runx2), zinc finger transcription factor (Osterix), muscle segment homeobox 2 (MSX2) (Qi et al., 2019), and is associated with increased proliferation and migration ability (Kapustin and Shanahan, 2016). The osteoblast-like phenotype of VSMCs is regarded as the cellular characteristic factor of vascular calcification. Many factors such as oxidative stress damage, hyperphosphatemic environment, and inflammation increase the indices related to bone formation in VSMCs and promote their transformation into osteoblasts (Voelkl et al., 2018a). On the other hand, a variety of biochemical factors are involved in the phenotype switching of VSMCs, i.e., the growth factors platelet-derived growth factor (PDGF-BB) and transforming growth factor-β1 (TGF-β1) could promote the phenotype switching of VSMCs (Jain et al., 2021).

Matrix vesicles (MVs), one kind of extracellular matrix-derived EVs, are membrane-bound microparticles released by cells, containing various cargo, including proteins, carbohydrates, lipids, DNA and mRNAs, and species of small RNAs, such as microRNAs (miRNAs). The origin and composition of MVs determine their calcification potential (Zazzeroni et al., 2018). Recent evidence showed that extracellular MVs serve as nucleating foci to initiate microcalcification (Demer and Tintut, 2014). MV nanofragments obtained by mechanical rupture could induce rapid mineralization *in vitro* (Kunitomi et al., 2019). The formation and secretion of MVs and the increase of intracellular alkaline phosphatase (ALP) activity are also involved in the phenotypic transformation and osteoblast-like phenotype transformation of VSMCs (Lin et al., 2016). However, the specific mechanisms and functions of MVs regulating vascular calcification have not been fully elucidated. For example, what is the originating cell that releases MVs in vascular calcification, and how do the pro-calcification MVs get into the recipient cell? On the other hand, increasing evidence shows that EVs have potential therapeutic applications for many diseases. For example, overexpressing a high-affinity variant human PD-1 protein (havPD-1) EVs has been used to reduce cancer cell proliferation and induce apoptosis (Chen et al., 2022). Another therapeutic role of EVs is dependent on their proteins and/or non-coding RNAs, in particular miRNAs delivery ability (de Abreu et al., 2020). It has also been shown that MVs carry miR-199a-3p, which, by regulating GATA-binding 4 acetylation, were able to rescue electric function in engineered and *in vivo* atria (Aday et al., 2017). Alginate-based microreactors that were loaded with MVs as support for bone-like osteoblast cells were shown to enhance biomineralization in a co-assembled 3D spheroid (Itel et al., 2018). Therefore, engineered MVs have emerged as a potential therapeutic method to treat vascular calcification. In this review, targeted interventions based on MVs to treat vascular calcification are also discussed (New et al., 2013).

## Vascular Calcification

**FIGURE 1:**
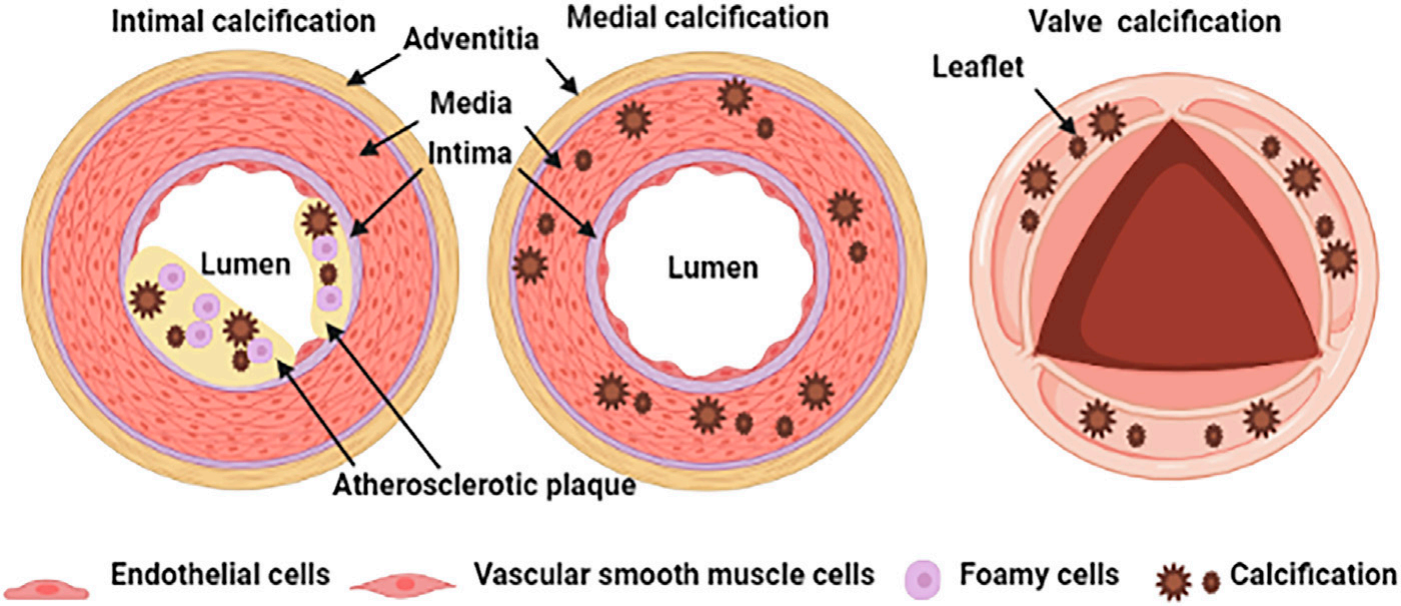
Schematic representation of vascular calcification classification.

Physiological calcification is a normal process that occurs in bones and teeth; however, pathological calcification occurs in soft tissue such as blood vessels, joints, and tumors, in association with aging, advanced atherosclerosis, diabetes, and chronic kidney disease (Jing et al., 2019). The classification of vascular calcification is divided into intimal calcification, media calcification and cardiac valve calcification Figure 1 (Viegas and Simes, 2018). Vascular intimal calcification often occurs in atherosclerotic diseases. Intimal calcification is related to the stability of atherosclerotic plaque and the microcalcifications that contribute to plaque rupture by concentrating mechanical forces within the fibrous cap (Blaser and Aikawa, 2018). After calcification is formed, it is scattered on the inner wall of the blood vessel in the form of a spot or patch (Nakahara et al., 2017). The early-stage shows microcalcification (range: ≥0.5 to <15 μm) and then develops a spot-like calcification (Nakahara et al., 2017). Vascular media calcification often occurs in the internal elastic lamina and is more common in small and medium arteries such as the femoral artery, tibial artery and radial artery, also known as Monckeberg’s medial sclerosis, which is more common in patients with advanced aging, diabetes and CKD (Zazzeroni et al., 2018). Medial calcification decreases vessel compliance, increases pulse wave velocity and systolic hypertension. The occurrence of cardiac valve calcification is related to mechanical stress and inflammation and is commonly seen in metabolic diseases such as diabetes, dyslipidemia, uremia, etc. When cardiac valve calcification occurs, spotted calcium salts are deposited, accompanied by valve fibrosis and inflammatory cell infiltration (Shekar and Budoff, 2018).

The main cause of vascular calcification is the ectopic deposition of hydroxyapatite crystals in blood vessel wall cells and extracellular matrix (ECM) (Viegas and Simes, 2018). During the last few decades, vascular calcification had been regarded as a process of accumulating insoluble precipitates of Ca phosphate without a specific cellular biological response. In recent years, increasing evidence shows that vascular cells actively regulate the calcification process (Proudfoot, 2019).

## The Contribution of Matrix Vesicles to Vascular Calcification

Many risk factors such as calcium and phosphorus metabolism disorder, inflammation, oxidative stress, apoptosis, autophagy and aging can contribute to vascular calcification. They can also intersect or interact with each other, thereby affecting the occurrence and development of vascular calcification (Pescatore et al., 2019). Vascular calcification is a major complication of CKD patients. This may be due to the decline of renal function, leading to calcium and phosphorus metabolism disorders, which in turn leads to hyperphosphatemia and hypercalcemia. Both hypercalcemia and hyperphosphatemia may promote intimal and media calcification. Hyperphosphatemia may increase the activity of the sodium-dependent cotransporters, PIT-1 and PIT-2, and up-regulate genes associated with matrix mineralization, which eventually leads to vascular calcification (Cozzolino et al., 2019). Interestingly, hypercalcemia and hyperphosphatemia both increase the release of MVs, leading to the deposition of hydroxyapatite in the extracellular matrix (Reynolds et al., 2004).

Inflammatory cells such as macrophages promote vascular calcification by secreting inflammatory cytokines such as TNF-a, IL-1β, and IL-6 (Henaut et al., 2016). Inflammatory cytokines directly promote calcification in VSMCs by down-regulating VSMC-specific genes and up-regulating osteoblastic genes such as BMP2 and ALP. They also indirectly reduce circulating Fetuin-A, MGP, and Klotho levels, further promoting vascular calcification procession in VSMCs (Henaut et al., 2016; Zhang et al., 2018). MVs secreted by pro-inflammatory cells also play pivotal roles in the procession of vascular calcification. These MVs participate in vascular calcification by forming the phosphatidylserine (PS)-annexin A5-S100A9 membrane complex involved in mineralization or by mediating the transport of miRNAs such as miRNA-153 and miRNA-223 (Henaut et al., 2016; Zhang et al., 2018).

Oxidative stress-induced by intracellular calcium and phosphate overloading promotes vascular calcification *via* inducing signal activation and VSMCs osteogenic phenotype transition (Nguyen et al., 2020). Pro-mineralizing MVs induced calcification of recipient VSMCs by stimulating reactive oxygen species (ROS) production (Chen et al., 2021). Apoptotic cells form a nidus for calcification, releasing apoptotic bodies loaded with calcium that participate in the calcification process by deposition of hydroxyapatite in the extracellular matrix (Cozzolino et al., 2019).

Emerging evidence has demonstrated that autophagy is directly related to vascular calcification. Crucially, autophagy maintains the physiological function of VSMCs by regulating Ca^2+^ homeostasis (Phadwal et al., 2020). Autophagy-related proteins and protein complexes play important roles in exosome biogenesis (Xu et al., 2018). It has been shown that MVs are rich in annexin-A5, which is also known to have an important role in the formation of mature autophagosomes (Ghislat and Knecht, 2012). Autophagic proteins including LAMP1, LAMP2, and LAMTOR1 were found in MVs released from rat VICs (Cui et al., 2016). In the progression of vascular calcification, MVs may be entwined with the network of autophagic vesicles during the formation or release stage (Cui et al., 2016). Cell autophagy has a special pro-calcification effect than cell apoptosis. It increases the inorganic phosphate (Pi)-induced MVs release with increased ALP activity (Dai et al., 2013). Therefore, inhibiting the autophagy pathway may help to prevent Pi-induced vascular calcification by reducing MVs release (Dai et al., 2013; Cui et al., 2016).

Vascular calcification is associated with aging, which is commonly seen in older adults or in middle-aged subjects affected by premature vascular aging. MVs released from senescent ECs and elderly subjects’ plasma promote calcification of VSMCs, and MV carriage of increased quantities of annexins A2 and A6, BMP2, and Ca (Alique et al., 2017). EVs released from senescent cells can also drive the senescence of neighboring cells in a paracrine manner (Blaser and Aikawa, 2018).

The above-mentioned studies underscore the essential role of MVs, which are totally dependent on their cellular source, in regulating the vascular calcification process. Therefore, the origin of MVs is briefly described below.

## The Origin of Matrix Vesicles

**FIGURE 2:**
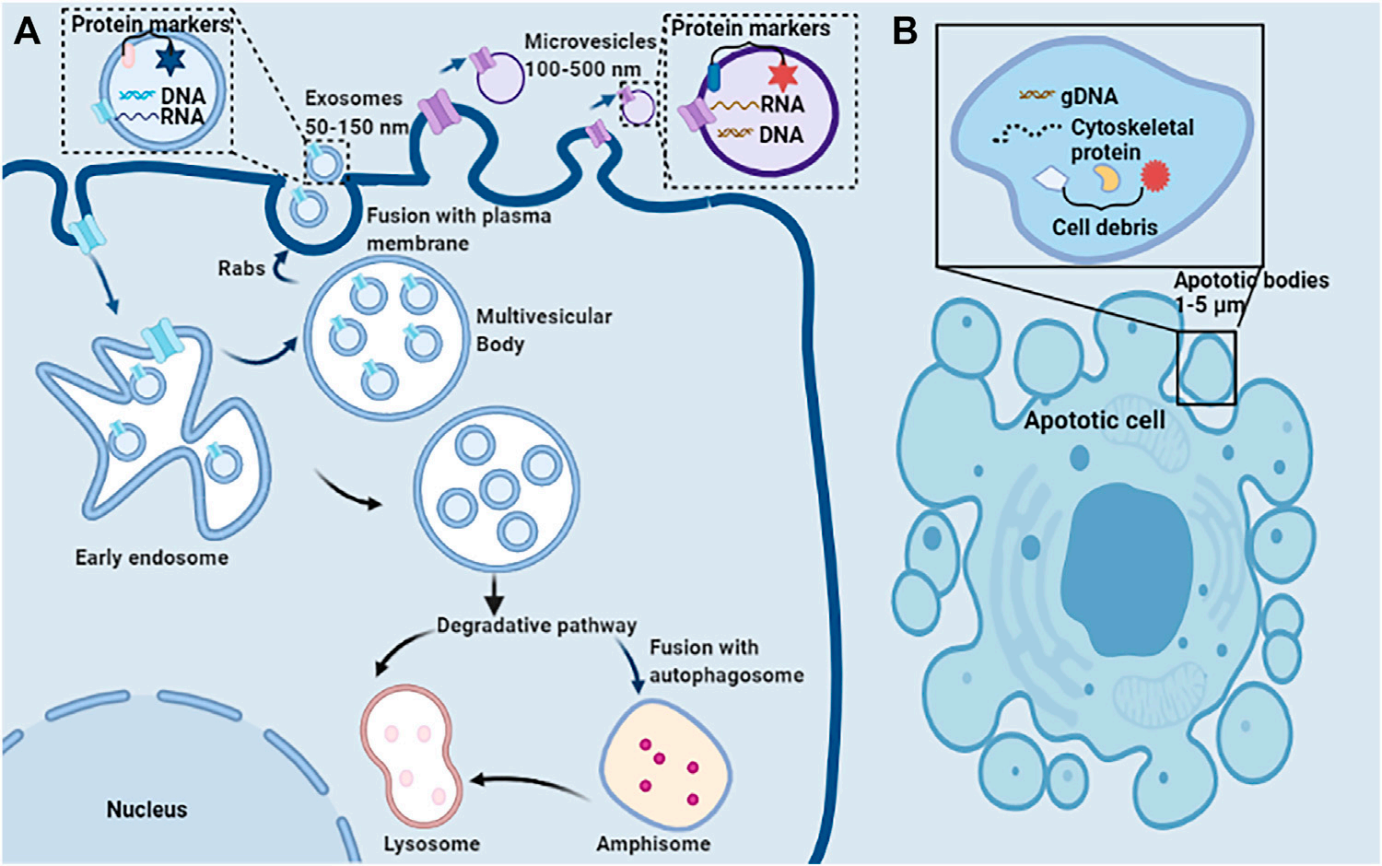
Schematic representation of the release, structure, and composition of extracellular vesicles. **(A)** Direct sprouting and splitting of the plasma membrane deformed into microvesicles (100–500 nm in diameter). In early endosomes, proteins are sequestered in intraluminal vesicles of the larger MVBs. The inward budding of endosomal membranes forms intraluminal vesicles of MVBs. MVBs bud inward and then fuse with the plasma membrane results in the release of their enclosed exosomes (∼50–150 nm) from the cell into the microenvironment. Due to its biophysical properties, MVBs can be degraded by entering the lysosome directly or fusing with autophagosome and then entering the lysosome. **(B)** Apoptotic or dying cells shrink to produce apoptotic bodies (ABs) (1,000–5,000 nm). ABs are condensed remnants of the apoptotic cell, with nuclear and cytoplasmic components.

EVs encompass a wide variety of vesicles with a lipid bilayer membrane structure released by various kinds of cells in either resting or stress states (Goettsch et al., 2013). The main characteristics of various EVs are described in Table 1. According to the route of biogenesis, size, density, and protein markers, EVs are categorized into exosomes (50–150 nm), microvesicles (100–500 nm), and apoptotic bodies (1,000–5,000 nm) (Mathivanan et al., 2010; Goettsch et al., 2013; Blaser and Aikawa, 2018) (Figure 2). Exosomes are released through the endosomal-sorting complex, where intraluminal vesicles formed by the inward budding of the endosomal membrane are packaged within multivesicular bodies (MVBs). These MVBs then fuse with the plasma membrane to release their enclosed exosomes in a Rab GTPase-dependent manner (Blaser and Aikawa, 2018). Microvesicles are formed by direct budding and fission of the plasma membrane (Mathivanan et al., 2010; Blaser and Aikawa, 2018). Apoptotic bodies are formed by apoptotic cells due to the breakdown of the cytoskeleton, which induces the plasma membrane to bulge, leading to the separation of the plasma membrane (Mathivanan et al., 2010; Blaser and Aikawa, 2018).

**TABLE 1:** Main characteristics of exosomes, matrix vesicles, microvesicles, and apoptotic bodies.

	Exosomes	Matrix vesicles	Microvesicles	Apoptotic bodies	References
Size	50–150 nm	30–300 nm	100–1,000 nm	1–5 μm	Blaser and Aikawa (2018)
Origin	Intraluminal vesicles within multivesicular bodies	Plasma membrane or intraluminal vesicles within multivesicular bodies	Plasma membrane and cellular content	Plasma membrane, cellular fragments	Shapiro et al. (2015), Stahl et al. (2019)
Mechanism of formation	Fusion of multivesicular bodies with the plasma membrane	Budding of the cell plasma membrane or fusion of multivesicular bodies with the plasma membrane	Outward blebbing of the plasma membrane	Cell shrinkage and programmed cell death	Shapiro et al. (2015), Stahl et al. (2019)
Release	Constitutive and/or cellular activation	Constitutive and/or cellular activation	Constitutive and/or cellular activation	Apoptosis	Stahl et al. (2019)
Pathways	Endosomal Sorting Complex Required for Transport (ESCRT)-dependent Tetraspanin-dependent Ceramide-dependent	ESCRT-dependent tetraspanin-dependent ceramide-dependent stimuli- and cell-dependent	Ca^2+^-dependent stimuli- and cell-dependent	Apoptosis-related	Stahl et al. (2019)
Markers	Tetraspanins (CD9, CD63, CD81,CD82, CD53, CD37, etc), MHC I, MHC II, Hsp70, Hsp90, Alix, TSG101, etc	Tetraspanins (CD9, CD81, CD63, etc), MHC I, LAMP-1, LAMP-2, TSG101, HSP90, HSP70, etc	Tetraspanins (CD40, CD83, CD31, CD34, etc), TSG101, phosphatidylserine, antigens from parent cells, ARF6, integrin VCAMP3, selectins, etc	Phosphatidylserine, propidum iodide positive, annexin V, TSP, C3b, caspase 3, histones, etc	Nabhan et al. (2012), Kapustin et al. (2015), Shapiro et al. (2015), Melki et al. (2017), Todorova et al. (2017), Zhang et al. (2017), Kubo (2018)
Contents	Proteins (MHC molecules, signal transduction, enzymes, etc.), nucleic acids (mRNA, microRNA, non-coding RNAs, ssDNAs, dsDNAs, etc.), lipids, etc	Proteins (MHC molecules, signal transduction, enzymes, etc.), nucleic acids (mRNA, microRNA, non-coding RNAs, ssDNAs, dsDNAs, etc.), lipids, etc	Nucleic acids (mRNA, microRNA, non-coding RNAs, ssDNAs, dsDNAs, mitochondrial DNA, etc.), cytoplasmic and membrane proteins (tissue factors, cytokines, enzymes, etc.), lipids, etc	Cell organelles, proteins, nuclear fractions, DNA, coding and non-coding RNA, lipids	Kubo (2018)
Functions	Progression, metastasis, and formation of the microenvironment of tumor, angiogenesis, antigen presentation, apoptosis, coagulation, cellular homeostasis, inflammation, intercellular signaling, etc	Sites of provisional mineralization	Pro-inflammatory and anti-inflammatory effects, cellular homeostasis, intercellular signaling, etc	Maintain homeostasis of the immune system, progression, metastasis, and formation of the microenvironment of tumor	Shapiro et al. (2015), Boulanger et al. (2017), Gurunathan et al. (2019)

In the process of vascular calcification, EVs that are released by macrophages are 30–300 nm in diameter and originate as membranous protrusions in the intima layer (Blaser and Aikawa, 2018). Besides this, VSMCs-derived EVs, with a diameter of 100–150 nm, originating from an exosomal pathway in the media layer, are processed by multivesicular bodies and are enriched in members of the Rab GTPase family (Blaser and Aikawa, 2018). Previous advances have shown that a common mechanism may mediate both intimal and medial vascular calcification, that is, a substantial increase of EVs in the vascular interstitial space, in particular, the small EVs (sEVs, with a size of 40–100 or to 140 nm) which are mainly produced and secreted from arterial VSMCs. These sEVs facilitate the formation of nucleate Ca phosphate (Ca/P) crystals in the form of Hydroxyapatite (Bhat et al., 2020a). Valve homeostasis also depends on appropriate intercellular interactions between valvular endothelial cells (VECs) and valvular interstitial cells (VICs) (Bakhshian Nik et al., 2017). EVs are released by VICs can influence VECs, and transfer of VIC-derived EVs containing perinuclear proteins to VECs cultures reveals intercellular vesicle transfer through the endosomal pathway (Krohn et al., 2016a; Bakhshian Nik et al., 2017).

MVs, another kind of EVs, are approximately 100–300 nm in diameter, membrane-invested particles, and can be located in the ECM (Bottini et al., 2018). Under physiological conditions, MVs can be released from hypertrophic chondrocytes, osteoblasts, odontoblasts, and tenocytes. In addition, some non-skeletal tissue cells such as VSMCs, in dead or dying macrophages, and VICs also release pathological MVs (Bakhshian Nik et al., 2017). Pro-vascular calcification MVs contain reactive oxygen species and pro-inflammatory cytokines. While inhibiting vascular calcification MVs secret anti-inflammatory factors and retards VSMCs to differentiate into osteoblast-like cells (Li et al., 2020). Macrophage-derived MVs express exosomal markers (CD9 and TSG101) and contain PS-annexin A5-S100A9 membrane complex, which promotes hydroxyapatite nucleation and directly contributes to microcalcification in chronic renal disease (New et al., 2013). MVs derived from macrophages enhance ectopic mineralization in the high Ca/Pi environment (Zhang et al., 2018). VSMCs-derived MVs ranging in size from 50 to 500 nm respond by disturbing intracellular calcium homeostasis and enhance mineralization by releasing the key mineralization inhibitor matrix Gla protein loading, enhancing matrix metalloproteinase-2 activity, and forming annexin A6/PS nucleation complexes (Kapustin et al., 2011). On the other hand, circulating EVs could be taken up by recipient VSMCs, which contribute to vascular calcification in CKD, through calcification/osteogenic differentiation and inflammatory status, leading to increased mineral deposition (Viegas et al., 2018).

In calcified aortic valve disease (CAVD), the phenotypic changes of VICs play a vital role in ECM remodeling and mineral deposition (Bakhshian Nik et al., 2017). Under pathological conditions, such as hyperphosphatemia, VICs can differentiate into two distinct phenotypes: an activated myofibroblast-like VIC (aVIC) or an osteoblast-like VIC (oVIC), which are responsible for the active deposition of Ca in CAVD (Hjortnaes et al., 2015). In this calcifying milieu, pro-calcific VIC-derived EVs are similar to MVs from chondrocytes and VSMCs, showing elevated annexins A2, A5 and A6 (Bakhshian Nik et al., 2017). In the standard Ca/Pi calcification environment, the expression of vascular calcification-associated protein annexin A6 and Ca content were significantly up-regulated in rat VICs derived MVs (Cui et al., 2017). Through bioinformatics analysis techniques, MVs from calcified VICs were analyzed to reveal calcification regulators and exosome markers, including CD9, CD63, LAMP-1, and LAMP-2 (Cui et al., 2017).

Based on the above content, MVs should not be confused with microvesicles according to their different biogenesis type, particle size, and functional location.

## Strategies and Opportunities to Modify Matrix Vesicles to Treat Vascular Calcification

### Nucleation and Crystallization of Minerals

Under pathological conditions, such as inflammation or hyperlipidemia, the increased expression and activity of ALP will promote calcification, stimulating the release of phosphate from biological sources (for example, ATP) for mineral nucleation (Blaser and Aikawa, 2018). In this process, the multiligand sorting receptor sortilin in EVs regulates the release of EVs and drives the activated ALP to load into calcifying EVs in a Rab11-dependent manner (Blaser and Aikawa, 2018). Importantly, MVs are a cell product that can be used as sites for hydroxyapatite crystal precipitation. The origin and content of MVs are essential factors in determining their mineralization potential (Kapustin et al., 2011). MVs can obtain mineral compounds to maintain intracellular mineral metabolic homeostasis and participate in the process of vascular calcification by promoting mineral deposition sites formation (Zhang et al., 2018). Elevated extracellular Ca and cytosolic Ca alterations are necessary to produce the mineralization-competent MVs (Kapustin et al., 2011). In the presence of high Ca, VSMCs can release more mineralization-competent MVs, which are filled with microspicules of crystalline mineral, predominantly composed of Ca, which would lead to matrix mineralization (Reynolds et al., 2004). Unlike the cell membrane composition, MVs are rich in annexins, sphingomyelins, and PS, which are vital mineral nucleation sites on the MVs membrane (Genge et al., 2007). Upon the stimulation of Ca and phosphate, calcification inhibitor is suppressed, and annexins/PS nucleation complexes is formed, providing hydroxyapatite nucleation sites (Kapustin et al., 2011; New et al., 2013). Meanwhile, the MVs membrane also contains calcification inhibitors such as fetuin-A and MGP, which prevent mineral nucleation (Reynolds et al., 2004). In calcifying EVs, the homeostasis between calcification inhibitors and promoters is broken, resulting in a loss of mineralization inhibition.

### MiRNAs in Matrix Vesicles Regulate the Process of Vascular Calcification

For the reason that the lipid bilayer of EVs can protect the small RNAs from degradation by extracellular ribonuclease and proteases (Blaser and Aikawa, 2018), small RNAs such as ribosomal RNA (rRNA), miRNAs, and transfer RNA (tRNA) are enriched in EVs. Interestingly, the total miRNA contained in EVs is higher, while the total RNA is less than the donor cells (Chaturvedi et al., 2015). MiRNAs in EVs could be transported and regulate the proliferation and differentiation of recipient cells. Furthermore, it also mediates intercellular communication during the vascular calcification process (Goettsch et al., 2013). In uraemic rats EVs, the expression of miR-221 and miR-222 were significantly enhanced, and the levels of miR-143 and miR-145 were significantly reduced. These EVs can participate in the phenotypic switch and the calcification of VSMCs *via* enhanced AKT signaling and PiT-1 expression (Freise et al., 2021). Endothelial cell-derived EVs are selectively loaded with miR-126, miR-143, and miR-145, which participate in VSMCs phenotype regulation by mediating paracrine signaling across cells within the vessel wall (Krohn et al., 2016a). Bone marrow mesenchymal stem cells derived exosomes inhibited high P-induced calcification in VSMCs through modifying miRNA profiles. The mTOR, MAPK, and Wnt signaling pathways were involved in this process (Guo et al., 2019). On the other hand, many miRNAs in EVs are also involved in inhibiting the function of vascular calcification. MiR-30, miR-125-b, miR-143, miR-145 and miR-155 loaded into calcifying VSMCs-derived EVs can influence the expression of a specific set of osteogenic markers such as Smad1, RUNX-2, ALP, and osterix, and change in the concentrations of these miRNAs in EVs lead to shifts in Ca and MAPK signaling pathways implicated in SMC-mediated calcification (Krohn et al., 2016a). Melatonin-induced exosomes miR-204 and miR-211 inhibit the osteogenic differentiation of VSMCs by reducing RUNX2 and BMP2 expression and ALP activity (Xu et al., 2020). Exosome-derived miR-324-3p regulate the expression levels of the IGF1R, MAP2K1 and PIK3CA proteins in the mouse smooth muscle cell line (MOVAS-1) and inhibit coronary artery calcification (Pan et al., 2020).

In the mineralization environment, especially the vascular calcification process, some miRNAs in MVs facilitate deposition of Ca orthophosphate, which can change Ca phosphate structure from an amorphous form to more crystalline structures, such as hydroxyapatite; some miRNAs have the opposite effect, inhibiting mineral deposition (Krohn et al., 2016a). In VMSCs from CKD rats, miR-667, miR-702, miR-3562, miR-3568, and miR-3584 are highly concentrated in MVs (Chaturvedi et al., 2015). Some miRNAs contained in MVs can regulate the phenotype of recipient cells. These studies indicate that the dysregulation of miRNAs in MVs may promote vascular calcification by causing abnormal mineral deposits, promoting hydroxyapatite’s formation, or by driving the transformation of VSMCs phenotype from a contractile to a synthetic and osteochondrogenic phenotype. MVs also act as carriers to transfer dysregulated miRNAs from cell to cell to induce changes in the function of the recipient cells, which leads to vascular calcification.

### Proteins Associated With Vascular Calcification in Matrix Vesicles

MVs contain a large number of proteins, each of which has specific characteristics and performs its specific functions in mineral deposits. The endogenous protein in MVs could be divided into three groups, inhibitors, promoters, and other proteins. The inhibitors in MVs, such as MGP, Gla-rich protein (GRP), and fetoglobulin A (fetuin-A) (Kapustin et al., 2015), suppress the calcification, while the promoters in MVs such as annexin family, sortilin, and S100 protein family aggregate the calcification. Finally, other proteins functionally regulate MVs secretion.

### Inhibitors in Matrix Vesicles

MGP is a 15-kDa vitamin K-dependent mineral binding protein, which is widely expressed in VSMCs and chondrocytes (Bjorklund et al., 2020). MGP is known as an effective inhibitor of Ca phosphate precipitation, which can inhibit aortic and cartilage calcification (Xiao et al., 2021). The reason why MGP exerts an anti-calcfication role is that carboxylated and phosphorylated MGP could eliminate the calcification effect of various bone morphogenetic proteins (BMPs) such as BMP-2 and BMP-4 (Houben et al., 2016; Bjorklund et al., 2020; Khan et al., 2021). In addition, MGP-fetuin-A complex inhibits ectopic mineralization by combining with alkaline Ca phosphate crystals (Xiao et al., 2021). MGP is a potent inhibitor of ECM mineralization; deficiency of MGP initiates arterial calcification (Khavandgar et al., 2014). Deposits of hydroxyapatite minerals were shown in the arterial wall of MGP-deficient (*Mgp* ^−/−^) mice, which died within 2 months (Khavandgar et al., 2014). The anti-calcification function of MGP is most likely related to the formation of MVs (Bjorklund et al., 2020). The elevated Ca concentration decreases the MGP expression in MVs, while a similar result is not observed upon high concentration of phosphate (Houben et al., 2016).

GRP (Gla-rich protein) is an inhibitor of vascular calcification involved in Ca homeostasis, and γ-carboxylated GRP could inhibit calcification and osteochondrogenic differentiation by α-SMA upregulation and osteopontin downregulation (Viegas et al., 2015). GRP was expressed at protein and mRNA levels in EVs released by macrophages, which may act as vehicles for ECM transport and information transmission (Viegas et al., 2017). It prevents Ca-induced signaling pathways and directly binds to minerals to inhibit crystal formation (Viegas et al., 2015). Ucma/GRP (Upper zone of growth plate and cartilage matrix-associated protein/Gla-rich protein) can directly interact with BMP2 and regulate phosphate-induced mineralization of VSMCs by participating in BMP-2-SMAD1/5/8 osteo/chondrogenic signaling pathway *in vitro* (Willems et al., 2018). GRP can form a large complex with MGP and fetuin-A, which is loaded in noncalcified EVs; the ratio of the complex is decreased in the high Ca-loaded vesicles (Xiao et al., 2021). Additionally, they can also combine with minerals to form a fetuin-mineral complex called colloids calciprotein particles (CPP), which greatly affects the stability of minerals (Xiao et al., 2021). Circulating CPPs and EVs, which contain lower levels of fetuin-A and GRP, are determinants of vascular calcification in CKD, with the capacity to modulate VSMCs responses through increased osteochondrogenic differentiation and inflammation, leading to increased mineral deposition (Viegas et al., 2018).

Fetuin-A is a plasma glycoprotein from the family of cystatin protease inhibitors with a TGF-β cytokine-binding motif and a Ca phosphate-binding site that can inhibit the precipitation of basic calcium phosphate *in vitro* (Khan et al., 2021). Fetuin-A is one of the biomarkers acting as calcification inhibitors, and it plays an important role in ectopic calcification. The possible mechanisms of fetuin-A involved in ectopic vascular calcification include inhibiting spontaneous mineral nucleation and growth and inhibiting the formation of hydroxyapatite crystals; combining clusters of Ca and phosphate to stabilize these ions and preventing cellular uptake; mediating the formation of protein mineral CPP (Icer and Yildiran, 2021). Fetuin-A can be loaded into MVs, which maintains the phenotype of healthy VSMCs and also binds minerals and stabilizes them against further growth, preventing vesicles from mineralizing (Chen and Moe, 2015; Kapustin et al., 2015).

Osteoprotegerin (OPG) is known to be a soluble tumor necrosis factor (TNF) superfamily receptor that inhibits the actions of the cytokine receptor activator of nuclear factor kappa-B ligand (RANKL) (Montanez-Barragan et al., 2014). OPG was also detected in VSMCs- derived MVs and co-located with annexin A6 (Cui et al., 2016). Under physiological conditions, OPG can be secreted by vesicles released from viable or apoptotic VSMCs and directly inhibit VSMCs mineralization by limiting MVs driven mineral nucleation and hydroxyapatite deposition in the vascular wall (Cui et al., 2016).

### Promoters in Matrix Vesicles

As the membrane factors of MVs, the annexin family members A2, A5, and A6 mediate Ca influx into MVs and are well-known calcification promoters (Kapustin et al., 2011). The massive enrichment of various annexins in calcifying MVs may be a key factor in regulating MVs release and calcification potential in the cardiovascular system. For example, annexin A2 could bind to fetuin-A at the cell membrane of VSMCs in the presence of high calcium and contribute to MVs-mediated calcification. Additionally, the high level of annexin A2 might also be one of the reasons that end-stage renal disease-exosomes promote VSMCs calcification (Lin et al., 2020). Annexin A6 was enriched in calcifying VIC-derived MVs, and these MVs may promote aortic valve calcification in end-stage renal disease (Cui et al., 2017). Most importantly, annexin A6, the largest member of the annexin family, is present highly in MVs. Biochemical analyses revealed annexin A6 could have three different localizations in MVs during physiological mineralization. They are Ca^2+^-bound annexin A6 interacting with the inner leaflet of the MVs membrane; annexin A6 localized on the surface of the outer leaflet; and annexin A6 inserted in the membrane’s hydrophobic bilayer and co-localized in lipid domains enriched in cholesterol (Veschi et al., 2020). Moreover, annexin A6 and Ca induced PS exposure on the MVs surface and formed of annexin A6/PS nucleation complexes, thus providing hydroxyapatite nucleation sites (Hjortnaes et al., 2015).

Sortilin is a sorting receptor that directs target proteins, including growth factors, signaling receptors, and enzymes, to the secretary or endocytic compartments of cells. Sortilin expression levels are atypically high in calcified arteries in CKD and atherosclerosis disease. Moreover, sortilin deficiency reduces vascular but not skeletal calcification *in vivo,* indicating that sortilin might be a key regulator of vascular calcification (Goettsch et al., 2016). Sortilin plays a direct role in ectopic calcification and has become a potential cardiovascular risk biomarker and a drug target for cardiovascular disease (Gebraad et al., 2018). In older men aged ≥50 years, higher serum sortilin levels are associated with a higher risk of major adverse cerebrovascular and cardiovascular events (MACCE) and severe abdominal aortic calcification (AAC) (Goettsch et al., 2017). The receptors for AGEs (RAGE) down-regulated sortilin and mediated the formation of microcalcification, whereas galectin-3 up-regulated sortilin and induced macrocalcification (Sun et al., 2019).

Sortilin could traffic and load the calcification protein TNAP into VSMC-derived EVs, conferring its calcification potential (Itoh et al., 2018). Sortilin forms homodimers in the extracellular and intracellular domains with intermolecular disulfide bonds, which likely facilitate the trafficking of sortilin to the EVs (Itoh et al., 2018). Sortilin could accelerate the formation of MVs aggregates in the early stage of calcification and affect the microcalcification signals (Sun et al., 2019). In a diabetic apolipoprotein E-deficient background (*ApoE* ^−/−^) mouse model, mouse tail vein injection of Nε-Carboxymethyl-lysine (CML)-induced MVs originated from VSMCs-derived obviously aggravated diabetic atherosclerotic calcification (Jing et al., 2020). High concentrations of CML significantly promoted the release of MVs from VSMCs and the recruitment of sortilin to MVs (Jing et al., 2020).

The S100 protein family has 21 members, which can be released from monocytes, VSMCs, and ECs in response to cellular stress stimuli. Some members of the S100 proteins family, such as S100A8, S100A9, and S100A12 have homologous structures and functions, commonly known as S100/calgranulins, and are closely related to cardiovascular disease (Xiao et al., 2020). These proteins bind with their receptors, such as advanced glycation end products (RAGE), scavenger receptors (CD36), and toll-like receptor 4 (TLR-4), contributing to the cellular response in vascular inflammation, vascular oxidative stress, and vascular calcification progression (Xiao et al., 2020). Among the S100 protein family, S100A9 is closely related to cardiovascular calcifying MVs. S100A9 is the Ca^2+^-binding neutrophil cytosolic protein, which is strongly expressed in calcifying areas, the surrounding extracellular matrix, and calcifying MVs (McCormick et al., 2005). S100A9 in atherosclerotic plaque and calcifying MVs may be involved in the pathogenesis of atherosclerosis and dystrophic calcification by influencing redox- and Ca^2+^-dependent processes (McCormick et al., 2005). Macrophage S100A9 plays an important role in vascular calcification in diabetes mellitus. When exposed to high glucose, pro-inflammatory macrophages-derived calcific EVs through the S100A9-RAGE axis contribute to the excessive microcalcification formation within plaques (Kawakami et al., 2020).

MMP-2, also known as gelatinase A, was involved in the process of vascular calcification. MMP-2 facilitates vascular calcification by catalyzing matrix degradation (Kapustin and Shanahan, 2016). The active form of MMP-2 is also found in VSMCs derived MVs, and elevated levels of extracellular Ca can induce enhanced MMP-2 activity. Inhibiting the activity of MMP-2 loaded in MVs can prevent vascular calcification (Kapustin and Shanahan, 2012).

Transglutaminase 2 (TG2) is a Ca-dependent enzyme that can facilitate cell-ECM interaction through integrins (Chen et al., 2013). TG2 becomes the core of inducing the arterial calcification program by promoting VSMCs transdifferentiation to osteoblasts and chondrocytes (Johnson et al., 2008). The expression of TG2 is increased in both VSMCs and MVs isolated from CKD rats. The cross-linking of the ECM with TG2 leads to enhanced cell-ECM and MVs-ECM attachment, induced phenotypic switch of VSMCs to contractile “osteoblast-like” cells, and reduced function of calcification inhibitors (Chen et al., 2013).

The phosphatase orphan 1 (PHOSPHO1), contained in MVs, as an additional inorganic phosphate (Pi) supplier was involved in the first step of MVs-mediated initiation of mineralization (Kiffer-Moreira et al., 2013; Bobryshev et al., 2014).

### The Regulator Controlling Matrix Vesicles Secretion

Sphingomyelin phosphodiesterase-3 (SMPD3) is not only a key signal molecule in the process of biomineralization but also a regulator of exosomal biogenesis. In VSMCs, elevated extracellular Ca increased SMPD3 expression. In turn, the up-regulation of SMPD3 could regulate exosome secretion and fetuin-A recycling (Kapustin et al., 2015). Increased ceramide in cell membrane or cytoplasm through the SMPD3 pathway may induce sEVs biogenesis, initiating arterial calcification (Bhat et al., 2020a). Lysosomal overexpression of *Smpd1* gene specifically in VSMCs may reduce lysosome-MVB interactions, which may reduce lysosome degradation of MVBs and increase the fusion of MVBs with the plasma membrane to release sEVs, and consequently leading to medial arterial calcification (Bhat et al., 2020a).

High mobility group box 1 (HMGB1), a nuclear protein that binds to chromatin, is usually released into the extracellular space as a damage-associated molecular pattern (DAMP) when cells are activated, damaged, or die (Chen et al., 2017). HMGB1 is also a cytokine associated with biological mineralization and could induce macrophages to secrete MVs through the RAGE/p38MAPK/nSMase2 signaling pathway to enhance ectopic mineralization (Chen et al., 2016). BMSC-derived exosomes alleviate high phosphate-induced aortic calcification and ameliorate vascular function *via* the SIRT6–HMGB1 deacetylation (Wei et al., 2021).

Microtubule stabilization inhibited hyperphosphatemia-induced vascular calcification by down-regulation of osteogenic signal and attenuation of MVs release (Lee et al., 2014). *Mucolipin 1* Gene deletion in mice increased sEVs secretion by inhibiting lysosomes and MVB interactions in arterial stiffening and medial calcification. The expression of exosome/sEVs markers, CD63, and annexin A2 were significantly increased in the coronary arterial wall of *Mcoln1* ^*−/−*^ mice (Bhat et al., 2020b). Endoplasmic reticulum (ER) stress induces calcification of VSMCs *in vitro* and modifies VSMCs phenotype by increasing the release of Grp78-loaded calcifying EVs (Furmanik et al., 2021).

The interaction between EVs and ECM accelerates the extracellular accumulation and aggregation of calcifying EVs. In human atherosclerotic plaque formation, a large amount of disorganized collagen will accumulate before calcification begins. However, the content of collagen is inversely proportional to the size of microcalcifications: when collagen is degraded, EVs can aggregate, nucleate hydroxyapatite and form microcalcifications (Blaser and Aikawa, 2018). Many high levels of collagen-binding proteins such as ALP, proteoglycan link proteins, hyaluronic-acid binding regions, and annexins are also found in calcifying EVs (Blaser and Aikawa, 2018). It indicated that the collagen protein inhibits the accumulation of EVs. The MVs secreted by VSMCs can locate close to collagen fibrils and interact with collagen type I (COL1) in VSMCs under calcifying conditions (Tyson et al., 2003). Fetuin-A, MGP, S100A9, and annexins have emerged as active regulators of this interaction (Reynolds et al., 2004). 8-degree-polymerized oligogalacturonic acid prevents vascular calcification development by inhibiting the osteogenic marker expression and dissociating the direct interaction between MVs and COL1 (Hodroge et al., 2017). In particular, this interaction can be inhibited by masking the GFOGER sequence, which reveals the specific areas on the collagen fibers that bind to EVs (Krohn et al., 2016b; Hodroge et al., 2017). The collagen receptor discoidin domain receptor-1 (DDR-1) can regulate collagen deposition and release of calcifying EVs by VSMCs through the TGF-β pathway. In calcification media, *DDR-1* ^*−/−*^ VSMCs increased the release of TGF-β1, which can stimulate P38 phosphorylation and inhibit the activation of SMPD3, thereby promoting EVs-mediated calcification and collagen matrix production (Krohn et al., 2016b).

### The Signal Pathway Involved in Matrix Vesicles Regulating Vascular Calcification

During the vascular calcification process, the production and release of cellular MVs is the first step, while the uptake of MVs by recipient cells is also essential. The detailed molecular mechanisms regulating MVs secretion and inclusions in vascular calcification are quite complicated. For instance, uraemic EVs augment Ca- and phosphate-induced osteogenic transdifferentiation of VSMCs *via* activated AKT and ERK signaling and PiT-1 expression (Freise et al., 2021). The lysosomal sphingolipid/ceramide pathway may be crucial participants in the secretion of sEVs and phenotypic switch in arterial VSMCs (Bhat et al., 2020a). Warfarin is known to be an oral anticoagulant and an effective inducer of calcification, which could increase EVs release and VSMCs calcification *via* the PERK-ATF4 ER stress pathway (Furmanik et al., 2021). The autophagy pathway may be a key participant in the regulation of MVs-mediated vascular calcification progression (Dai et al., 2013). Through bioinformatic analysis using Ingenuity Pathway Analysis (IPA) revealed the up-regulation of key signaling pathways crucial to cardiovascular function in calcifying VIC-derived MVs in end-stage renal disease, including aldosterone, P2Y purinergic receptor signaling, and thrombin signaling pathways, Rho signaling, and metal binding (Cui et al., 2017). Cellular-derived MVs with characteristics of exosomes and low fetuin-A content from calcifying VSMCs of rats with CKD enhanced the calcification of recipient VSMCs by inducing cell signaling changes and phenotypic switch of recipient VSMCs (Chen et al., 2018). Several major cell signaling pathways were involved in this process: 1) mitogen-activated protein kinase (MAPK) signaling specifically, increased MEK1 and ERK signaling; 2) increased intracellular Ca from sarcoplasmic reticulum stress; 3) increased NADPH oxidase (NOX) and superoxide dismutase (SOD) activity; 4) activation and increased intracellular Ca, leading to reducing VSMCs calcification (Chen et al., 2018). EVs secreted from both patients with acute coronary syndrome and senescent ECs induce early senescence of recipient endothelial cells and cellular oxidative stress. MAPK, Akt, and p53 signaling pathways may be involved in this process (Blaser and Aikawa, 2018).

## Toolbox: Isolation and Validation of Matrix Vesicles

### Ultracentrifugation

UC is the most commonly used method for MVs purification at present. Differential centrifugation can be used to separate vesicles of similar sizes. UC is usually used in conjunction with sucrose density gradient centrifugation to separate low-abundance small vesicles (Hutcheson et al., 2014). Although this combined method can obtain highly purified MVs, it also has some disadvantages: the steps are complicated with an inconsistent recovery rate. Most importantly, repeated centrifugation is likely to disrupt vesicles and reduce their quality. Some soluble proteins may contaminate the sample by forming clumps with the vesicles, potentially impacting proteomic and RNA content analysis (Lobb et al., 2015).

### Ultrafiltration

MVs can also be obtained by selective separation of samples with ultrafiltration membranes of different pore sizes. Compared to UC, UF is less time-consuming and does not require the use of special equipment (Cheruvanky et al., 2007). The combination of UF and size exclusion chromatography (UF/SEC) is superior to the traditional UC method in terms of production time, standardization, scalability, and vesicle yield. Compared with traditional UC, using UF/SEC, approximately 400 times more small extracellular vesicles (sEVs) per ml of media were recovered, and upscaling this process further increases sEVs yield by about 3-fold (Cardoso et al., 2021).

### Magnetic Bead Sorting

CD63, CD31, CD9, 70 kDa heat shock proteins (HSP70), and tumor susceptibility gene 101 (TSG101) are the well-known marker genes of MVs. After the MVs are incubated with magnetic beads coated with anti-label antibodies, they can be adsorbed and separated. Using a process of immunoprecipitation based on polymeric beads that were modified with an antibody for tissue nonspecific alkaline phosphatase (TNALP), MVs were isolated from an organotypic culture model (Iordachescu et al., 2018). The magnetic bead sorting method has the advantages of high specificity, simple operation and can maintain the complete shape of MVs (Kluszczynska et al., 2019). However, antigen-antibody binding requires sufficient time for action, so this method generally has the problem of time-consuming operation and low extraction efficiency (Lane et al., 2017), and it may also be affected by the pH value and salt concentration of the separation solution.

### Polymer-Based Precipitation

Because of its simplicity and feasibility, polymer-based precipitation such as polyethylene glycol (PEG) precipitation has gained popularity. After incubating with PEG at 4°C, the MVs are precipitated and recovered by filtration or centrifugation. Although this method is effective in obtaining small EVs (sEVs), some of the non-sEV contaminants are also precipitated at the same time (Nakai et al., 2016). Therefore, this method is not commonly used due to the shortcomings of low vesicle recovery rate, low purity, and uneven particle size of isolated MVs (Lobb et al., 2015).

In recent years, various extraction kits have been used for the isolation of MVs. These kits are increasingly popular because of their simple operation, high extraction efficiency, and the ability to extract urine, blood, cell supernatant, and other samples separately. Researchers have developed other methods, including HPLC (high-performance liquid chromatography)-based protocols (Zeringer et al., 2015), affinity-capture methods (Iordachescu et al., 2018), microfluidic-based isolation of vesicles methods (Wang et al., 2013), acoustic-based purification methods (Lee et al., 2015), etc. Each of these separation methods has its unique advantages, but their disadvantages also limit their wide use.

## The Identification Methods of Matrix Vesicles

### Nanoparticle Tracking Analysis

NTA is one of the most commonly used methods to count the number of MV particles. NTA can analyze the size of small vesicles particles in batches and obtain a particle size distribution curve (Hoshino et al., 2020). It is easy to operate and can protect the structure and function of MVs from damage. However, the concentration of the sample particles greatly influences the results. Therefore, to obtain stable and reliable results, the sample needs to be diluted to an appropriate concentration.

### Protein Marker Detection

Enzyme-linked immunosorbent assay (ELISA), flow cytometry and western blot can be used to detect MVs protein markers. Commonly used markers include CD31, CD9, CD63, annexins, integrin receptors, TSG101 and HSP70 (Shapiro et al., 2015).

### Quantitative Real Time-Polymerase Chain Reaction Analysis

qRT-PCR is the most sensitive and reliable method to detect gene expression. MVs contain a large number of microRNAs and these microRNAs play an important role in the formation of mineral nucleation sites and the osteogenic differentiation of VSMCs. qRT-PCR is an indispensable method for studying these microRNAs (Sohn et al., 2015). At the same time, a bioanalyzer and droplet digital PCR(ddPCR) can be used to detect the RNA quality and quantity of MVs load.

### Electron Microscopy

Transmission electron microscopy (TEM) is a standard morphological detection method for MVs. The particle size of MVs can be observed by TEM, and the MVs are spherical and cup holder-like structures (Greening et al., 2015). Now some laboratories are also beginning to use atomic force microscopes to look at the shape of vesicles. Density-dependent scanning electron microscopy (SEM) can image calcifying EVs *in-situ*, and assess their size, density, and biophysical accumulation in calcified tissues, hydrogels, or matrices (Blaser and Aikawa, 2018). With the help of super-resolution microscopy, it is possible to obtain *in situ* imaging of vesicles using confocal light-based approaches (Blaser and Aikawa, 2018).

### Immunogold Labeling

Immunogold labeling is a kind of immunolabeling technology that uses colloidal gold as a tracer to participate in the antigen-antibody response. Exosomes were incubated with primary antibody and the secondary antibody conjugated to gold particles and then observed under an electron microscope after washing and counter staining (Hannafon et al., 2016). It has the advantages of simple and quick operation, high sensitivity, and stable markers.

## Discussion

**FIGURE 3:**
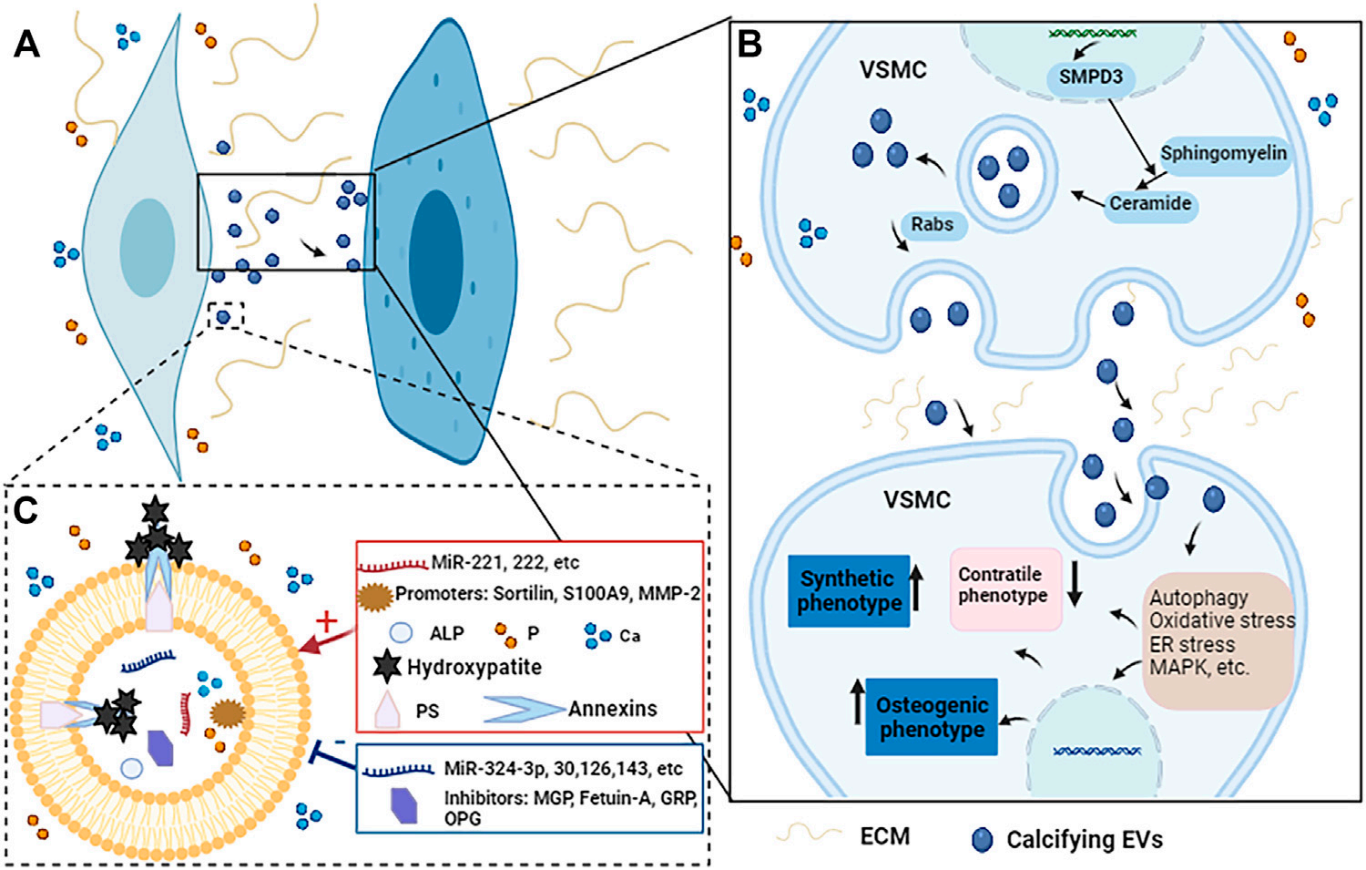
The potential regulatory mechanisms of calcifying EVs regulate the phenotypic switch and osteogenic differentiation of VSMCs during vascular calcification. **(A)** When VSMCs are exposed to elevated Ca and P conditions, the calcifying EVs it releases act on the recipient VSMCs to change their phenotype and biological function. **(B)** Under pro-calcific milieu, SMPD3 can enhance the secretion of exosomes, which are secreted in a Rabs-dependent manner. Calcifying EVs can regulate the phenotypic transformation and osteogenic differentiation of recipient VSMCs through multiple signaling pathways such as autophagy, oxidative stress, ER stress, and MAPK, etc. **(C)** Moreover, the composition of calcifying EVs determines its potential to load hydroxyapatite. When EVs are exposed to high Ca and P conditions, the balance between calcification promoters and inhibitors in EVs is broken, increasing the content of promoters such as Ca, P, microRNAs, and annexins. At the same time, the concentration of some inhibitors such as MGP, fetuin-A, and some microRNAs is reduced. Therefore, the mineral concentration gradient between the intra- and extra-vesicular spaces drives the influx of P and Ca into EVs *via* suitable transporters to form mineral nucleation sites. The annexins and PS complexes provide hydroxyapatite nucleation sites, leading to calcifying EVs and microcalcification.

Vascular calcification is a complex, active and highly regulated biological process. As important transporters for material transport and intercellular communication, MVs are closely related to vascular calcification. MVs can participate in vascular calcification by promoting the phenotypic transformation of VSMCs, regulating mineral deposits, mediating microRNAs transport, regulating cell signaling pathways (Figure 3).

MVs are endogenous nanovesicles secreted by living cells. Therefore, they are more suitable as therapeutic drug carriers due to their advantages of small particle size, low toxicity, non-immunogenicity, good permeability, and high targeting. Recent studies have shown that VSMCs-derived MVs have shown positive results in many disease models, such as reducing the size of myocardial infarction and improving the inflammation caused after myocardial infarction, resolving pulmonary hypertension, ameliorating renal fibrosis, and restoring neurovascular functions and plasticity (Blaser and Aikawa, 2018). In addition to using MVs as delivery vehicles, the contents of calcifying EVs themselves have also been proven to be an essential source of drug targets (Blaser and Aikawa, 2018). Therefore, MVs may be a new treatment for vascular calcification.

Additionally, researchers have successfully used exosomes to carry small molecules, nucleic acids, and proteins to treat various diseases in recent years. It may be possible to use exogenous engineered EVs as a viable therapeutic strategy for vascular calcification, whereby inhibitors of vascular calcification could be efficiently conveyed into atherosclerotic plaques or vascular microcalcification areas. However, the use of EVs as a nano-drug delivery system is still in its infancy in treating diseases, and researchers are still facing many problems and challenges, such as the low rate of drug encapsulation in EVs (Gaurav et al., 2021). So far, there is not a feasible method to treat vascular calcification. Some potential medications that address vascular calcification have been studied only in preclinical setups or lack extensive clinical research. Potential medications and treatment methods for addressing vascular calcification are listed in Table 2. The role of MVs in the occurrence and development of vascular calcification has not been clearly studied. We have not fully understood, under pathophysiological conditions, how the effective or key molecules in MVs change. This may be due to the disrupted balance between the promoters and inhibitors, the content of mirRNA-30, 125b, 126, 143, 145, fetuin-A, MGP, and GRP that inhibit calcification in MVs is reduced; in contrast, mirRNA-221, 222, annexins, MMP-2 are increased. How do the effective molecules interact with each other? For example, PS exposed on the outside of MVs membrane can bind to GRP and interact with annexins to form complexes that determine the formation of nucleation sites. How are the effective molecules packaged into the MVs? MVs are small in size, easy to penetrate biofilm, and lipid bilayer membrane structure can protect the small RNAs and proteins in EVs from degradation by extracellular ribonuclease and proteases. MVs can enrich signal molecules and make them have a higher local concentration. How are MVs generated and released? SMPD3 is a key protein regulating MVs production. The small Rab GTPases such as Rab11, Rab27, Rab35 regulate MVs secretion by different ways mediating the transport of MVBs toward the plasma membrane. How are the targeted transport of MVs regulated, and how do target cells recruit and activate MVs? This may be because in pathological environments, MVs can induce receptor-mediated signal transduction *via* surface-binding ligands; deliver specific functional proteins and miRNAs to target cells through cells surface receptors. In order to find out the specific treatment methods of vascular calcification based on MVs for vascular calcification in future studies, we can focus on mineralization-related proteins and miRNAs in MVs, and we could use MVs as carriers to develop safer and more effective nanomedicines.

**TABLE 2:** Potential medications and treatment methods to treat vascular calcification.

Potential medications and treatment methods	References
Piperlongumine	Shi et al. (2020)
Sevelamer	Drueke and Floege (2020)
Sodium thiosulfate	Omarjee et al. (2020)
SNF472, the hexasodium salt of phytate	Perello et al. (2020)
Denosumab and bisphosphonates	Florea et al. (2020); Goody et al. (2020)
Inositol phosphates derivatized with ethylene glycol oligomers	Schantl et al. (2020)
Mitoquinone	Cui et al. (2020)
Biguanide (Metformin), dipeptidyl Peptidase-4 inhibitors, sulfonylureas, sodium glucose cotransporter-2 inhibitors, thiazolidinediones, insulin, alpha glucosidase inhibitors	Ghosh et al. (2020)
ATP-based therapy	Villa-Bellosta (2019)
Teniposide	Liu et al. (2018)
Estrogen, growth hormone-releasing hormone and its agonist	Shen et al. (2018)
Fibulin-3	Luong et al. (2018)
Phosphate binders, statins, vitamin K	Florea et al. (2020)
Zinc sulfate	Voelkl et al. (2018b)
Locking with ^18^F-NaF and loading with vitamin K	Florea et al. (2020)
Intravascular lithotripsy	Brinton et al. (2019)
